# Improved Pharmacokinetics and Tissue Uptake of Complexed Daidzein in Rats

**DOI:** 10.3390/pharmaceutics12020162

**Published:** 2020-02-16

**Authors:** Anna Kwiecień, Jana Ruda-Kucerova, Kamil Kamiński, Zuzana Babinska, Iwona Popiołek, Krzysztof Szczubiałka, Maria Nowakowska, Maria Walczak

**Affiliations:** 1 Department of Inorganic and Analytical Chemistry, Jagiellonian University Medical College, Faculty of Pharmacy, Medyczna 9, 30-688 Kraków, Poland; anna.kwiecien@uj.edu.pl; 2 Department of Pharmacology, Faculty of Medicine, Masaryk University, Kamenice 5, 625 00 Brno, Czech Republic; jkucer@med.muni.cz (J.R.-K.); zuzana.babinska@sukl.cz (Z.B.); 3 Faculty of Chemistry, Jagiellonian University, Gronostajowa 2, 30-387 Kraków, Poland; gawlikiwona2.O@gmail.com (I.P.); k.szczubialka@uj.edu.pl (K.S.); maria.nowakowska@uj.edu.pl (M.N.); 4 Chair and Department of Toxicology, Jagiellonian University Medical College, Faculty of Pharmacy, Medyczna 9, 30-688 Kraków, Poland

**Keywords:** daidzein, γ-cyclodextrin, bioavailability, nonlinear pharmacokinetics, tissue uptake

## Abstract

The pharmacokinetic profile and tissue uptake of daidzein (DAI) was determined in rat serum and tissues (lungs, eyes, brain, heart, spleen, fat, liver, kidney, and testes) after intravenous and intraperitoneal administration of DAI in suspension or complexed with ethylenediamine-modified γ-cyclodextrin (GCD-EDA/DAI). The absolute and relative bioavailability of DAI suspended (20 mg/kg i.v. vs. 50 mg/kg i.p.) and complexed (0.54 mg/kg i.v. vs. 1.35 mg/kg i.p.) was determined. After i.p. administration, absorption of DAI complexed with GCD-EDA was more rapid (t_max_ = 15 min) than that of DAI in suspension (t_max_ = 45 min) with a ca. 3.6 times higher maximum concentration (C_max_ = 615 vs. 173 ng/mL). The i.v. half-life of DAI was longer in GCD-EDA/DAI complex compared with DAI in suspension (t_0.5_ = 380 min vs. 230 min). The volume of distribution of DAI given i.v. in GCD-EDA/DAI complex was ca. 6 times larger than DAI in suspension (38.6 L/kg vs. 6.2 L/kg). Our data support the concept that the pharmacokinetics of DAI suspended in high doses are nonlinear. Increasing the intravenous dose 34 times resulted in a 5-fold increase in AUC. In turn, increasing the intraperitoneal dose 37 times resulted in a ca. 2-fold increase in AUC. The results of this study suggested that GCD-EDA complex may improve DAI bioavailability after i.p. administration. The absolute bioavailability of DAI in GCD-EDA inclusion complex was ca. 3 times greater (F = 82.4% vs. 28.2%), and the relative bioavailability was ca. 21 times higher than that of DAI in suspension, indicating the need to study DAI bioavailability after administration by routes other than intraperitoneal, e.g., orally, subcutaneously, or intramuscularly. The concentration of DAI released from GCD-EDA/DAI inclusion complex to all the rat tissues studied was higher than after administration of DAI in suspension. The concentration of DAI in brain and lungs was found to be almost 90 and 45 times higher, respectively, when administered in complex compared to the suspended DAI. Given the nonlinear relationship between DAI bioavailability and the dose released from the GCD-EDA complex, complexation of DAI may thus offer an effective approach to improve DAI delivery for treatment purposes, for example in mucopolysaccharidosis (MPS), allowing the reduction of ingested DAI doses.

## 1. Introduction

Daidzein (DAI) (7-hydroxy-3-(4-hydroxyphenyl)-4H-chromen-4-one) is a natural isoflavone present as a glucoside in leguminous plants, especially in soybean plant. DAI exhibits a variety of beneficial effects on human health; therefore, it is a compound of great clinical and pharmacological interest. Its broad therapeutic activities include cardioprotective [1], anticancer (prostate, ovary, colon, lung, breast, bladder) [2,3,4,5,6,7,8], anti-allergic [9], antidiabetic [10], anti-inflammatory [11], and anti-oxidative [11,12] effects. Since it shows structural similarity to estrogens [13], it can be used as a drug for estrogen replacement therapy to prevent and treat osteoporosis [14,15,16]. DAI can cross the blood–brain barrier [17] and shows cognition-enhancing effects [18], decreases anxiety and aggression, and increases locomotor activity in mice [19]. Importantly, DAI inhibits the cellular synthesis of glycosaminoglycans (GAGs). This is why DAI and other isoflavones like genistein are considered possible drugs for the treatment of mucopolysaccharidoses (MPS), and of Sanfilippo syndrome (MPSIII) in particular [20,21,22,23,24,25,26]. A phase III clinical trial is ongoing with the aim of finding out whether high doses of genistein are beneficial in MPSIII [27]. The therapeutic potential of isoflavones to treat MPSIII is of particular interest because to date, there is no drug available for this rare, lethal disease, with survival age ranging from 15 to 23 years [28].

However, low solubility of DAI in water, low oil/water partition coefficient, and intensive metabolism in the intestine and liver [29] result in its low bioavailability, drastically limiting its pharmacotherapeutic potential [30]. The PK profiles of DAI administered as a single-bolus dose [31] or in multiple doses [32] in healthy premenopausal women indicated that bioavailability of DAI was low and nonlinear at higher intakes (5.15% vs. 8.7%) suggesting that DAI uptake was rate-limited and saturable.

To increase DAI bioavailability and, consequently, to enhance its therapeutic effects, a number of approaches have been applied. One of them is derivatization with water soluble (ionizable) groups, e.g., sulfation [33], phosphation [34], or glucosylation [35]. However, besides being challenging to synthesize, these derivatives are still not soluble enough and/or show poorer biological activity than unmodified DAI. The second approach is to encapsulate DAI and related compounds in polymeric nanoparticles composed of, for example, hypromellose [36], carboxymethyl cellulose [37], zein [38], Eudragit E100–polyvinyl alcohol [39], poly(D,L)lactic acid [40], polymeric dendrimers [41,42], and polymeric microparticles composed of gelatin [43] or chitosan [44]. The third common approach is inclusion complexation of DAI with different cyclodextrins (CDs) and their derivatives, which was found to significantly improve its bioavailability, cellular membrane penetration, and therapeutic properties [45,46,47,48]. Importantly, DAI and genistein complexed with β-CD decreased GAG levels in the fibroblasts of MPSII and MPSIII patients [49].

In our previous studies, we obtained ethylenediamine-modified γ-cyclodextrin (GCD-EDA) and its inclusion complex with DAI (GCD-EDA/DAI). We found that the complex easily penetrated human fibroblasts and murine hippocampal neuronal cells [50]. Moreover, we found that DAI delivered in the form of GCD-EDA/DAI complex decreased the total GAG levels in normal human dermal fibroblasts [50]. These encouraging results prompted us to extend our studies on the delivery of DAI by applying the above complex to an in vivo rat model. We investigated the pharmacokinetic (PK) profile of DAI administered intravenously (i.v.) or intraperitoneally (i.p.), in suspension or in the form of GCD-EDA/DAI complex, to rats. To measure the concentration of DAI in serum and tissue homogenates, an original method using an LC-ESI/MS/MS system was developed and validated.

## 2. Materials and Methods

### 2.1. Materials

Daidzein (>98% purity), genistein used as an internal standard (IS) (>98% purity), formic acid of LC/MS purity, and PBS buffer (pH = 7.4) were purchased from Sigma-Aldrich (St. Louis, MO, USA). Methanol and acetonitrile of HPLC purity were supplied by Merck (Darmstadt, Germany) and ethylenediamine (EDA)-modified γ-cyclodextrin complex with daidzein (GCD-EDA/DAI) was synthesized as previously described [50]. The DAI content in the complex was 2.7%. Ultra-pure water was obtained using a Milli-Q system from Millipore (Bedford, MA, USA).

### 2.2. Apparatus

The LC-ESI/MS/MS experiments were performed on an Applied Biosystems/MDS Sciex (Concord, ON, Canada) API 2000 triple quadrupole mass spectrometer equipped with an electrospray ionization interface. This instrument was coupled to an Agilent 1100 (Agilent Technologies, Waldbronn, Germany) LC system. Data acquisition and processing were accomplished using the ABSciex Analyst 1.4.2 data collection and integration software. The chromatographic separation was performed on a Hypersil GOLD analytical column (100 mm × 3 mm i.d., 5 μm, Thermo Scientific, Waltham, MA, USA) with the column temperature set at 30 °C.

### 2.3. Standard Solutions, Calibration Curves, and Quality Control Samples

The stock solution of DAI was prepared in methanol at a concentration of 0.5 mg/mL, and serial dilutions of the stock solution in the same solvent down to the working DAI concentrations of 0.25, 0.5, 1, 2.5, 5, 10, 25, 50, and 100 ng/mL for the calibration curve (CC) were prepared. The quality control (QC) samples were prepared at three concentration levels: low QC, 7.5 ng/mL; medium QC, 40 ng/mL; and high QC, 75 ng/mL.

Calibration samples were prepared by spiking 90 µL of rat serum or tissue homogenates with 10 µL of a suitable working solution of DAI. The spiked samples were then vortexed for 3 s, and protein precipitation was performed by adding 200 µL of the methanolic solution of the IS at a concentration of 125 ng/mL and mixing for 20 min. All standard curves consisted of seven to nine concentration levels and calibration samples were freshly prepared for each of three analytical runs. The best fit of standard curves for all analytes was obtained applying the 1/x weighing algorithm.

### 2.4. Sample Preparation

Protein precipitation with methanol was used for purification of serum and tissue homogenates. The frozen serum and tissue samples were thawed at ambient temperature. An amount of 200 mg tissue weighed on an analytical balance was homogenized with an electric tissue homogenizer in 1 mL of PBS buffer. A volume of 100 µL serum or tissue homogenate was transferred to 2 mL Eppendorf tubes and 200 µL methanolic solution of IS at a concentration of 125 ng/mL was added for the precipitation of proteins and mixed for 20 min, followed by centrifugation (28,672× *g*) for 10 min at 4 °C. The supernatant was transferred to chromatographic vials and 20 µL was injected into the analytical column.

### 2.5. LC/MS/MS Analyses

The chromatographic separation was performed using the mobile phase composed of eluent A—HPLC grade acetonitrile acidified with 0.1% (v/v) formic acid, and eluent B—HPLC grade water with 0.1% (v/v) formic acid. The elution gradient started with 90% of eluent B, increasing to 90% of eluent A over 5 min, returned to 90% of eluent B over 5 min, and maintained at 90% of eluent B for 5 min. The mobile phase flow rate was set at 400 µL/min. The injection volume was 20 µL and the total time of analysis was 15 min.

For increased sensitivity and selectivity, the MS/MS data acquisition was performed in the selected reaction monitoring (SRM) mode. The ions measured were *m/z* 255.1 (Q1) and *m/z* 199.1 (Q3) for DAI, and *m/z* 271.1 (Q1) and *m/z* 215.2 (Q3) for IS.

The optimized MS/MS experimental conditions were as follows: ion spray voltage: 5500 V; source temperature: 200 °C; gas 1 pressure: 30 psi, gas 2 pressure: 40 psi, curtain gas pressure: 20 psi, collision gas pressure: 12 psi. Nitrogen (99.9%; from a nitrogen generator produced by Peak Scientific Instruments Ltd., Fountain Crescent, Inchinnan Business Park, Inchinnan, PA4 9RE, Scotland, UK) was used as the curtain and collision gas. The optimized MS/MS conditions used for analysis of the target analytes are shown in Table 1.

### 2.6. Method Validation

The developed method was validated according to validation procedures, parameters, and acceptance criteria based on FDA and EMA guidelines for bioanalytical method validation [51,52].

### 2.7. Treatment Groups and Sample Collection

A group of 66 male, 8 week old, albino Sprague–Dawley rats, weighing between 300 and 325 g each, were purchased from Charles River Laboratories (Germany) and housed in standard polycarbonate cages, in groups of four animals per cage. Environmental conditions during the study were constant: relative humidity 50%–60%, temperature 23 °C ± 1 °C, normal 12-h light–dark cycle (6 a.m. to 6 p.m. light). Standard rodent chow and water were available ad libitum.

GCD-EDA/DAI inclusion complex was dissolved in saline and administered by an i.v. injection at a dose of 20 mg/kg in 2 mL (which corresponded to 0.54 mg/kg of DAI) and i.p. injection at a dose of 50 mg/kg in 2 mL (which corresponded to 1.35 mg/kg of DAI). DAI in the form of a suspension in saline was administered to the rats at the doses of 20 mg/kg (i.v.) and 50 mg/kg (i.p.).

In the first study, 18 animals were deeply anaesthetized by i.p. injections of 50 mg/kg ketamine plus 8 mg/kg xylazine, and DAI in suspension (n = 9) or in GCD-EDA/DAI complex (n = 9) was administered i.v. to the tail vein. Blood samples were collected via a retro-orbital puncture at 15 min, 30 min, 3 h, 5 h, and 24 h after i.v. administration. The first two blood samples (at 15 min and 30 min) were withdrawn while the rats were still under general anesthesia. For the later time-points, the blood was taken under a short isoflurane anesthesia. One mL of saline was administered i.p. after every blood withdrawal to compensate the loss of water. The rats were sacrificed by decapitation 24 h after compound administration. All blood samples were centrifuged to obtain serum. The serum samples were frozen in a freezer at −80 °C.

The second study was conducted in 48 animals to assess DAI tissue penetration and bioavailability. DAI in suspension or in the GCD-EDA/DAI complex was administered i.p. and the animals were sacrificed at specific time-points: 15 min (*n* = 6), 45 min (*n* = 6), 3 h (*n* = 6), and 5 h (*n* = 6) after administration. Animals were deeply anaesthetized by i.p. injections of 50 mg/kg ketamine plus 8 mg/kg xylazine before sacrifice. First, the blood was collected by retro-orbital puncture, and the blood samples were centrifuged to obtain serum. The serum samples were frozen in a freezer at −80 °C. A whole-body saline perfusion was then performed after wide laparotomy when the heart was exposed, and the tip of the needle was inserted into the left ventricle and the right atrium was cut to allow efflux of blood. This procedure allowed the blood from all tissues to be washed out to determine the amount of DAI in the tissue, excluding its content in the blood. Following the animals’ euthanasia, the following samples were collected: whole brain, whole eyes, liver lobe, abdominal fat, one kidney, whole spleen, one testis, and selected parts of heart and lungs. The brain was cut between hemispheres in halves; one half was weighed, inserted into 2 mL of SDS-tris lysing buffer (100 mM tris buffer, 10% glycerol, 1% sodium dodecyl sulfate), and homogenized with a tissue homogenizer. The other half of the brain, the homogenates, and all other tissue samples collected were immediately shock-frozen in liquid nitrogen and stored at −80 °C for future analysis.

### 2.8. Pharmacokinetic Calculations

The PK profile of DAI was investigated after i.v. or i.p. administration in suspension or in GCD-EDA/DAI complex. PK parameters were calculated using a non-compartmental approach from the average concentration values, using Phoenix WinNonlin software (Certara, St. Louis, MO, USA). The first-order elimination rate constant (λ_z_) was calculated by linear regression of log concentration versus time. The area under the mean serum and tissue concentration versus time curve (AUC_0⟶_*_t_*) was calculated from zero to the last concentration point using the linear trapezoidal rule as

(1) $${\mathrm{AUC}}_{0\to t}=\overset{n}{\underset{i=1}{\sum }}\frac{C_{i}+C_{i+1}}{2}\cdot \left(t_{i+1}-t_{i}\right)$$

where $C_{i}$ is the concentration of the compound.

The area under the first-moment curve (AUMC_0⟶_*_t_*) was estimated by calculation of the total area under the first-moment curve:

(2) $${\mathrm{AUMC}}_{0\to t}=\overset{n}{\underset{i=1}{\sum }}\left((t_{i}\cdot C_{i}+t_{i+1}\cdot C_{i+1})/2\right)\cdot \left(t_{i+1}-t_{i}\right)$$

where $t_{i}$ is the time of the last sampling.

Mean residence time (*MRT*) was calculated as

(3) $$MRT=\frac{AUMC_{0\to t}}{AUC_{0\to t}}$$

Systemic clearance (*Cl*) was calculated as

(4) $$Cl=\frac{D_{iv}}{AUC_{0\to t}}$$

The volume of distribution at steady state (*V_ss_*) was calculated as

(5) $$V_{ss}=\frac{D_{iv}\cdot AUMC_{0\to t}}{{\left(AUC_{0\to t}\right)}^{2}}$$

The absolute bioavailability of DAI (*F*(%)) after *i.p.* administration was calculated as

(6) $$F\left(\%\right)=\frac{AUC_{i.p.}}{AUC_{i.v.}}\cdot \frac{D_{i.v.}}{D_{i.p.}}\cdot 100$$

where *D_i.v._* and *D_i.p._* are i.v. and i.p. doses of DAI in suspension or in GCD-EDA/DAI complex, respectively.

The relative bioavailability of DAI after i.p. administration in suspension or in the form of GCD-EDA/DAI complex was calculated as

(7) $$F_{rel}\left(\%\right)=\frac{AUC_{B}}{AUC_{A}}\cdot \frac{D_{A}}{D_{B}}\cdot 100$$

where *D_A_* and *D_B_* are i.p. doses of DAI in suspension or in GCD-EDA/DAI complex, respectively.

### 2.9. Statistical Analyses

All data are expressed as means ± SEMs (standard error of the mean). Statistical analysis was performed using GraphPad Prism 6.0. The differences in PK parameters among groups were obtained with the aid of Student’s *t*-test. For each tissue, AUC_0→t_ and SEM were calculated for both formulations. A *p* value of <0.05 was considered significant.

## 3. Results and Discussion

The main purpose of this study was to find out whether complexation of DAI might improve its poor bioavailability and thereby enhance its numerous therapeutic effects. We were also interested to find out whether the complex passes the blood–brain barrier (BBB), which would be of particular interest in the treatment of Sanfilippo disease (mucopolysaccharidosis type III, MPSIII), which severely affects the central nervous system.

As a delivery vehicle for DAI, we used a cationic derivative of γ-cyclodextrin (GCD) synthesized by substitution with ethylenediamine (EDA), as described previously [50]. We previously successfully used the GCD-EDA/DAI complex to deliver DAI to murine neuronal cells and human fibroblasts [50]. In the cytotoxicity studies on fibroblasts (human adult dermal fibroblasts (ATCC, PCS-201-012™), it was verified that GCD-EDA was completely non-toxic up to the concentration of 100 μg/mL [50]. This is of particular importance as the low toxicity of the DAI carrier is a prerequisite of its applicability, taking into account the quite low (2.7%) content of DAI in the material.

In the current paper, we present a continuation of the studies of DAI delivery in the form of GCD-EDA/DAI complex using an in vivo rat model. The complex was administered to the rats both i.v. (0.54 mg/kg) and i.p. (1.35 mg/kg). As a control, DAI administered in suspension (i.v.—20 mg/kg and i.p.—50 mg/kg) was used.

To quantitatively determine the concentration of DAI in serum and tissues, we developed an original LC/ESI-MS/MS method (see Supplementary Materials) which was successfully applied to estimate the PK profile of DAI, its absolute and relative bioavailability, and the distribution to brain, eye, fat, heart, liver, lungs, kidney, testes, and spleen.

In this study, a classic pharmacokinetic approach was used on four different occasions, each using different dose of DAI, in suspension and in GCD-EDA complex, in a single intravenous or intraperitoneal bolus. The PK parameters of DAI calculated using a non-compartmental approach are listed in Table 2.

After i.v. administration of DAI in suspension (20 mg/kg) or in the form of GCD-EDA/DAI complex (0.54 mg/kg), the concentration–time plots varied depending on the dose and formulation used. The initial concentration (C_0_) of DAI in suspension was ca. 100 times higher than in GCD-EDA/DAI complex; however, this relationship did not correlate linearly with the dose. Nevertheless, DAI in the GCD-EDA/DAI complex was eliminated ca. 2.5 times slower than DAI in suspension, so the half-life of DAI administered i.v. as the GCD-EDA/DAI complex was ca. 1.7 times longer. It should be noted that the formulation of DAI has a significant impact on the volume of distribution with the likelihood of penetrating to deep compartments and binding to tissues. Volume of distribution at steady state for DAI in GCD-EDA/DAI complex was ca. 6 times larger than for DAI in suspension.

**Figure 1:**
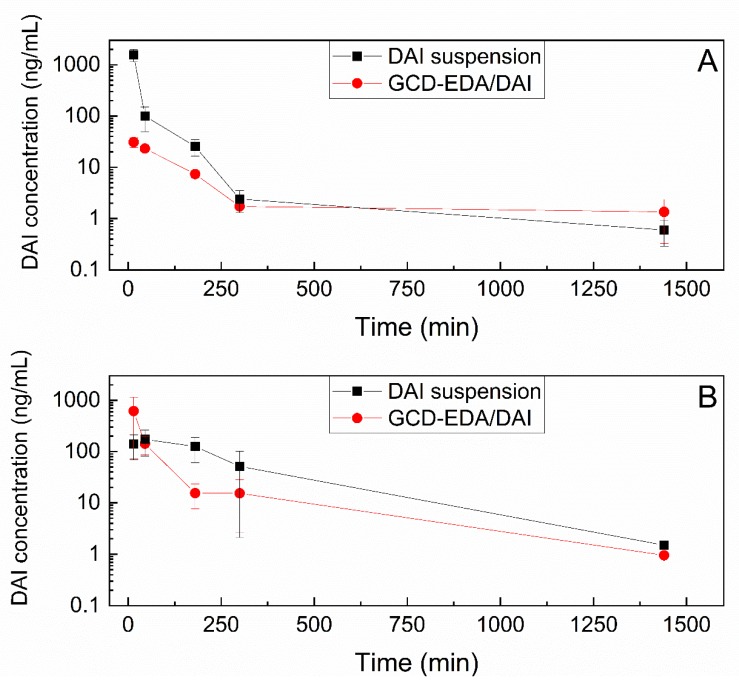
The dependence of DAI concentration in suspension or in GCD-EDA/DAI complex in serum on time after i.v. (**A**) and i.p. (**B**) administration to rats. Error bars show standard deviation.

After i.p. administration of DAI in a suspension (50 mg/kg) or in the form of GCD-EDA/DAI inclusion complex (1.35 mg/kg), the concentration–time plots were different depending on dose and formulation used (Figure 1B). DAI released from GCD-EDA/DAI complex reached the maximum concentration ($C_{\max}$) faster (within 15 min) compared to the suspension (within 45 min), and $C_{\max}$ reached a 3.4-fold higher value (614.7 ng/mL vs. 173.1 ng/mL). In humans, DAI is absorbed relatively quickly, attaining maximum serum concentrations at times ranging from 2 to 8 h after ingestion.

The clearly visible nonlinear pharmacokinetics can be seen in the nonlinear increase of AUC with increasing dose. The intravenous dose of DAI in suspension was 34 times higher than in GCD-EDA complex, which resulted in a 5-fold increase in AUC. In turn, the intraperitoneal dose of DAI in suspension was 37 times higher than in GCD-EDA complex, which resulted in a ca. 2-fold increase in AUC.

Despite the fact that the intravenous and intraperitoneal doses of DAI in the complex were 34 and 37 times lower, respectively, than the dose of the compound in the suspension, the absolute bioavailability of DAI in the complex was ca. 3 times higher (82.4% vs. 28.2%), whereas the relative bioavailability of DAI in the complex was ca. 21 times higher than in suspension.

**Figure 2:**
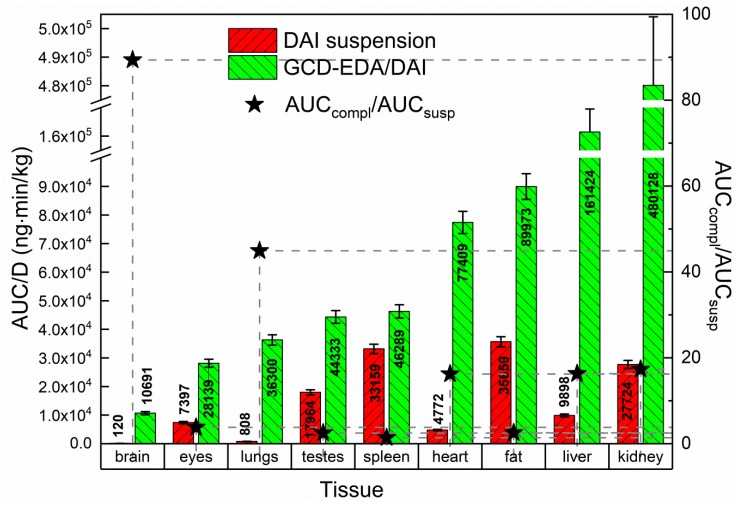
Tissue distribution of DAI after i.p. administration in suspension (50 mg/kg) or in GCD-EDA/DAI complex (1.35 mg/kg) in rats. The area under the curve was dose-normalized. The stars show the ratio of the AUC_0⟶t_ of DAI in each studied tissue after administration in GCD-EDA/DAI complex and in suspension.

Following the evaluation of the serum profile of DAI we determined its tissue penetration. Figure 2 illustrates the tissue uptake of DAI administered in suspension (50 mg/kg) and in GCD-EDA/DAI complex (1.35 mg/kg) after i.p. injection.

Substantial differences between the areas under the mean serum concentration versus time (AUC_0⟶t_) and normalized by the dose in tissues were observed (*p* < 0.05). The highest tissue uptakes of DAI after administration of its suspension and GCD-EDA/DAI complex were found in fat (AUC_0⟶t_/*D* = 35,659 ng·min/kg) and kidney (AUC_0⟶t_/*D* = 480,128 ng·min/kg), respectively; thus, the latter was about 13 times higher than the former. Moreover, significantly better uptake of DAI into all the tissues was found for complexed DAI compared to DAI in suspension. Importantly, DAI uptake by brain was enhanced the most (almost 90 times) when administered as a complex. This finding is of outstanding importance from the point of view of the possible application of complexed DAI in the treatment of Sanfilippo disease. The uptake by lungs was also significantly increased (about 45 times) followed by kidney, liver, fat, heart, spleen, and testes. The lowest concentration of DAI complexation was found in the eyes. Statistically significant changes in AUC_0⟶t_/D between DAI and GCD-EDA/DAI groups were observed in the following tissues: eyes, liver, fat, kidney, lungs, testes, and heart (*p* < 0.0001). No changes were noted in the spleen (*p* = 0.37).

The most striking observation related to the formulation intake was the lack of linearity between the AUC_0⟶t_ of the serum concentration–time profile and the dose. Rather than a doubling of the AUC_0⟶t_ with a doubling of the dose, a curvilinear relationship was obtained. Usually, within the dose-range in which the pharmacokinetics are linear, the AUC_0⟶t_/dose ratios should be more or less constant. The fact that these consistently decreased indicates nonlinear pharmacokinetics.

In the literature, there is a lot of variability and discrepancies on DAI bioavailability [31,32,53,54,55]. The bioavailability, as apparent from the AUC, showed a curvilinear relationship with increasing levels of isoflavones ingested. Taken together, the serum and urinary data confirmed that the pharmacokinetics of DAI is nonlinear. When the increase in AUC is higher than that predicted on the basis of a linear relationship with dose, it usually indicates that one or more elimination pathways, such as metabolizing enzymes or transporters (renal or biliary) are saturated. When the AUC increase is less than that expected on the basis of a linear relationship, it is indicative of either increased elimination, which is usually due to induction of metabolizing enzymes, or reduced absorption. In the case of DAI, absorption is more likely to account for the observed nonlinearity. Therefore, the reduced systemic bioavailability can be likely explained by reduced absorption of DAI with increasing levels of intake and saturable metabolism. This curvilinear relationship becomes more evident at even higher isoflavone intakes, as observed in studies of ^13^C daidzein given in the dose range 0.4–1.8 mg/kg body. Overall, these findings show that there may be a limited advantage to consuming high amounts of isoflavones in single doses, because absorption appears to be rate-limited [31].

## 4. Conclusions

Comparative PK studies of DAI administered in suspension and in the form of GCD-EDA/DAI inclusion complex by i.v. and i.p. routes in rats were carried out and support the concept that the bioavailability of DAI from suspension and cyclodextrin complex is nonlinear. The complexed DAI uptake by several tissues was significantly enhanced compared to suspended uncomplexed DAI. The brain penetration by complexed DAI was especially high when compared with DAI suspension (an almost 90-fold increase). This indicated that the GCD-EDA/DAI formulation may become a suitable delivery system for DAI and could be used in future studies on the therapeutic applications of DAI, particularly in the treatment of the Sanfilippo disease. However, whether this delivery system performs equally well when administered by other more practical routes, such as oral, intramuscular or subcutaneous, remains to be verified. Therefore, the next step of our studies will be to assess the bioavailability of DAI after oral gavage. Since low content of DAI in the material constitutes a significant limitation of the delivery system, we also plan to optimize the DAI content in the material by changing the substitution degree of GCD with EDA, and by modifying the synthesis procedure of the complex.

## 5. Statement on Ethical Commitments

All experiments were conducted in accordance with all relevant laws and regulations governing animal care. The experimental protocol was approved by the Committee on the Protection of Animals, Faculty of Medicine, Masaryk University and by the Committee of the Ministry of Agriculture of the Czech Republic, and it was carried out under the recommendations of the European Community Guide for the Care and Use of Laboratory Animals (Directive 2010/63/EU of the European Parliament and of the Council of 22 September 2010 on the protection of animals used for scientific purposes).

## Figures and Tables

**Table 1:** The ion transitions and the applied values of collision energy (CE), declustering potential (DP), focusing potential (FP), entrance potential (EP), and the collision cell exit potential (CXP) established for the analytes.

Compound	Transition	CE (V)	DP (V)	FP (V)	EP (V)	CXP (V)
DAI	255.1⟶199.1	50	60	300	10	10
IS	271.1⟶215.2	45	70	200	10	10

**Table 2:** In vivo pharmacokinetic parameters of DAI administered in suspension or in GCD-EDA/DAI inclusion complex after i.v. (*n* = 9 animals per time point) or i.p. (*n* = 6 animals per time point) administration to the rats.

Parameters	DAI in Suspension		GCD-EDA/DAI	
	i.v. (20 mg/kg)	i.p. (50 mg/kg)	i.v. (0.54 mg/kg)	i.p. (1.35 mg/kg)
$C_{0}$ (ng/mL)	6162	-	63.11	-
AUC_0⟶t_ (ng·min/mL)	94,667	66,703	18,348	37,790
t_0.5_ (min)	230	204.7	379.5	300.4
C_max_ (ng/mL)	-	173.1	-	614.7
t_max_ (min)	-	45	-	15
MRT (min)	257.3	217.7	299.4	126.5
V_ss_ (L/kg) * V_d_/F (L/kg)	6.2	* 220	38.6	* 15.3
Cl (mL/min/kg) * Cl/F (mL/min/kg)	210.8	* 774.6	84.1	* 35.3
F (%)	28.2		82.4	

$C_{0}$—initial concentration; AUC_0→t_—area under the curve; t_0.5_—elimination half-life; MRT—mean residence time; C_max_—maximum concentration; t_max_—time to reach C_max_; V_ss_—volume of distribution at steady state; V_d_/F—volume of distribution normalized on bioavailability; Cl—systemic clearance; Cl/F—systemic clearance normalized on bioavailability, *—after i.p. administration.

## Supplementary Materials

The following are available online at https://www.mdpi.com/1999-4923/12/2/162/s1, Table S1: Intra- and interday accuracy, precision, recovery, absolute and relative matrix effect for DAI in serum and tissues (*n* = 3), Table S2: Stability data for DAI in serum and tissues (*n* = 3); Figure S1. Estimation of elimination rate constants using non-compartmental analysis after i.v. (A), i.p. (B) administration of DAI suspension, and i.v. (C), i.p. (D) administration of GCD-EDA/DAI complex (3–5 points were used in calculation).
