# Supplementary material for: Improved Pharmacokinetics and Tissue Uptake of Complexed Daidzein in Rats
Source: Pharmaceutics. 2020 Feb 16;12(2):162. doi: 10.3390/pharmaceutics12020162 (PMC7076374; doi:10.3390/pharmaceutics12020162)
Supplement: Supplementary file 1 [file pharmaceutics-12-00162-s001.pdf]

pharmaceutics

Supplementary Materials: Improved Pharmacokinetics and Tissue Uptake of Complexed Daidzein in Rats

Anna Kwiecień, Jana Ruda-Kucerova, Kamil Kamiński, Zuzana Babinska, Iwona Popiołek, Krzysztof Szczubiałka, Maria Nowakowska and Maria Walczak

Method validation

The calibration curves were plotted as the relationship between the peak area ratios of analyte/IS and the nominal concentration of the analyte. Given that deuterated DAI standard is not commercially available, genistein was used as IS for the quantification analysis. The method showed good linearity over the concentration range of 0.25–100 ng/mL for lungs and eyes, 0.5–100 ng/mL for serum, brain, heart and testes, and 1–100 ng/mL for fat, liver, kidney and spleen.

The precision and accuracy of the method were estimated based on five replicates per every quality control (QC) samples including three concentration levels: 7.5 ng/mL, 40 ng/mL and 75 ng/mL. The interday precision and accuracy were assessed for three analytical runs analysed within three consecutive days. The validated method allows determining the concentration of DAI in plasma as well as in tissue homogenates with high precision and accuracy (Table S1).

Table S1. Intra– and interday accuracy, precision, recovery, absolute and relative matrix effect for DAI in serum and tissues ($n = 3$).

Tissue	Concentration (ng/mL) * (ng/g)	Accuracy (%)		Precision (%)		Recovery (%)	Absolute ME (%)	Relative ME (CV,%)
		Intraday	Interday	Intraday	Interday			
serum	7.5	101.9	100.7	4.2	3.8	88.5	88.6	4.8
	40	96.4	98.6	1.4	3.7	128.2		
	75	101.7	100.7	6.2	4.1	117.8		
brain	7.5	98.3	98.3	9.9	6.1	105.0	88.8	4.5
	40	103.2	103.4	1.9	1.2	79.1		
	75	98.4	98.4	3.6	2.4	94.2		
eye	7.5	101.3	102.1	2.4	8.2	113.3	86.4	6.8
	40	97.2	95.9	3.0	2.5	102.1		
	75	101.3	101.9	5.5	4.0	99.8		
fat	7.5	100.9	99.9	3.6	4.5	111.4	85.6	5.5
	40	98.2	100.2	1.8	2.6	113.8		
	75	100.8	99.9	3.5	3.4	85.4		
heart	7.5	100.4	99.9	6.9	7.3	115.9	87.6	4.2
	40	99.2	100.2	4.0	4.6	120.7		
	75	100.4	99.9	2.4	1.7	89.6		
liver	7.5	108.0	104.0	11.2	7.9	112.5	85.5	5.7
	40	98.7	98.2	2.2	3.0	109.0		
	75	100.6	100.8	6.6	4.4	106.9		
lung	7.5	99.5	100.0	11.0	8.3	113.5	83.03	4.7
	40	101.2	100.1	9.0	6.5	114.7		
	75	99.6	100.0	4.3	3.9	107.4		
kidney	7.5	100.4	99.7	6.4	5.6	111.7	87.2	5.6
	40	102.6	102.0	6.5	5.5	107.2		
	75	97.8	98.6	5.3	4.8	99.8		
testes	7.5	102.8	100.7	1.6	3.8	119.2	86.7	4.9
	40	101.3	101.7	12.4	7.2	115.7		
	75	102.4	100.5	2.9	3.2	98.7		
spleen	7.5	98.9	99.2	6.9	4.4	108.1	81.2	5.6
	40	102.2	101.5	4.4	2.9	109.0		
	75	103.4	101.1	10.2	6.6	95.3		

* concentration in tissues.

To evaluate the recovery, the peak area ratios of analyte/IS for serum and tissue homogenate samples spiked with standard solutions before the precipitation with those spiked after the precipitation were compared. The mean recovery was found to be within the range 79.1 ± 7% to 128.2 ± 7% (Table S1) indicating that sample preparation via precipitation is highly efficient and suitable for DAI purification from serum and tissue homogenates.

The method development and validation involved the assessment of the matrix effect, which may cause the alteration of the ionization efficiency in biological materials. To evaluate the absolute and relative matrix effect, QC samples were prepared using six different serum and tissue sample lots collected from rats. The absolute matrix effect was separately calculated for DAI for three concentrations (7.5 ng/mL, 40 ng/mL and 75 ng/mL) as the ratio between the analyte peak area obtained for samples spiked with a standard solution after matrix extraction and the analyte peak area registered for a standard solution. The normalized absolute matrix effect was calculated by dividing the absolute matrix effect of the analyte by the absolute matrix effect of IS. The highest mean values of the normalized absolute matrix effect of 88.8% were observed (Table S1). An absolute matrix effect lower than 100% indicates ionization suppression, whereas a value higher than 100% may suggest ionization enhancement. The relative matrix effect was expressed as the CV of the normalized absolute matrix effect calculated for six investigated matrix lots and as CV of standard line slopes plotted for the same matrices. In our study, the relative matrix effect calculated as the CV of the normalized absolute matrix effect did not exceed 15% of the permitted value.

The stability of DAI in biological samples depends on many factors, like the chemical properties of the molecule itself, the storage conditions and the matrix type. Rat serum and tissue samples were spiked with standard solutions to get concentrations of the final analytes of 7.5 ng/mL, 40 ng/mL and 75 ng/mL, frozen at −80 °C and analysed for short-term (7 days) and long-term (21 days) stability, stability in the autosampler, and after the three freeze–thaw cycles. To calculate the analytes' stability, the peak area ratio of analyte/IS obtained for thawed samples was compared with the peak area ratio of analyte/IS estimated for freshly prepared samples. The stability of DAI was assessed based on three measurements per concentration. The criteria for acceptability of the data include the accuracy within 85–115%

of the nominal values and the precision within ±15% of the relative standard deviation (RSD). The results have shown that DAI was stable in the studied matrices under the storage conditions with the accuracy of the experimental determinations greater than 90.7%, and the RSD less than 11% (Table S2).

Table S2. Stability data for DAI in serum and tissues (n = 3).

Tissue	Storage Conditions	QC 7.5 ng/mL			QC 40 ng/mL			QC 75 ng/mL		
		Mean ± SD [ng/mL]	RSD [%]	Accuracy [%]	Mean ± SD [ng/mL]	RSD [%]	Accuracy [%]	Mean ± SD [ng/mL]	RSD [%]	Accuracy [%]
serum	48 h at 4 °C in autosampler	7.57 ± 0.30	4	101	40.53 ± 1.32	3	101	73.73 ± 0.96	1	98
	three freeze - thaw cycles	6.99 ± 0.68	10	93	42.60 ± 3.12	7	106	70.23 ± 3.04	4	94
	frozen at −80 °C for 7 days	7.61 ± 0.25	3	101	39.33 ± 0.85	2	98	75.53 ± 168	2	101
	frozen at −80 °C for 21 days	7.82 ± 0.58	7	104	39.93 ± 2.12	5	100	76.73 ± 4.08	5	102
brain	48 h at 4 °C in autosampler	7.50 ± 0.37	5	100	41.43 ± 0.55	1	103	74.600 ± 0.700	0.9	99
	three freeze - thaw cycles	7.33 ± 0.63	9	98	41.70 ± 2.65	6	104	72.20 ± 3.99	5	96
	frozen at −80 °C for 7 days	7.28 ± 0.19	3	97	41.30 ± 0.10	0.2	103	74.03 ± 1.20	2	99
	frozen at −80 °C for 21 days	7.82 ± 0.31	4	104	41.10 ± 1.25	3	103	73.73 ± 2.38	3	98
eye	48 h at 4 °C in autosampler	7.22 ± 0.78	11	96	40.40 ± 1.54	4	101	76.60 ± 2.88	4	102
	three freeze - thaw cycles	7.33 ± 0.63	9	98	39.73 ± 1.81	4	99	72.20 ± 3.99	5	96
	frozen at −80 °C for 7 days	8.14 ± 0.11	1	108	37.73 ± 0.59	1	94	75.83 ± 1.90	2	101
	frozen at −80 °C for 21 days	7.73 ± 0.48	6	103	41.27 ± 1.55	4	103	76.10 ± 1.40	2	101
fat	48 h at 4 °C in autosampler	7.39 ± 0.38	5	99	38.87 ± 1.67	4	97	72.97 ± 1.23	2	97
	three freeze - thaw cycles	7.35 ± 0.38	5	98	39.66 ± 1.38	3	99	71.33 ± 1.75	2	95
	frozen at −80 °C for 7 days	7.56 ± 0.39	5	101	40.47 ± 1.07	3	101	75.63 ± 1.43	2	101
	frozen at −80 °C for 21 days	7.58 ± 0.33	4	102	39.90 ± 1.61	4	100	75.37 ± 2.30	3	100

**heart**	48 h at 4 °C in autosampler	7.37 ± 0.48	6	98	39.91 ± 3.26	8	100	72.97 ± 0.44	0.6	97
	three freeze - thaw cycles	7.53 ± 0.54	7	100	39.83 ± 1.80	4	99	71.77 ± 2.89	4	96
	frozen at −80 °C for 7 days	7.23 ± 0.57	8	96	39.90 ± 2.35	6	100	74.60 ± 1.18	2	99
	frozen at −80 °C for 21 days	7.07 ± 0.44	6	94	41.77 ± 2.72	6	104	72.33 ± 3.56	5	96
**liver**	48 h at 4 °C in autosampler	6.80 ± 0.27	4	91	39.60 ± 0.96	2	99	72.53 ± 1.46	2	97
	three freeze - thaw cycles	7.05 ± 0.55	8	94	39.27 ± 0.67	2	98	72.83 ± 1.66	2	97
	frozen at −80 °C for 7 days	7.57 ± 0.30	4	101	38.87 ± 1.70	4	97	76.10 ± 2.72	3	101
	frozen at −80 °C for 21 days	7.51 ± 0.47	6	101	41.27 ± 2.57	6	103	73.63 ± 3.59	5	98
**lungs**	48 h at 4 °C in autosampler	7.28 ± 0.32	4	97	36.30 ± 0.95	3	91	75.97 ± 4.04	5	101
	three freeze - thaw cycles	7.21 ± 0.07	1	96	38.10 ± 1.39	4	95	71.80 ± 1.91	3	96
	frozen at −80 °C for 7 days	7.65 ± 0.63	8	102	39.67 ± 2.57	6	99	75.83 ± 3.71	5	101
	frozen at −80 °C for 21 days	7.83 ± 0.12	2	104	37.43 ± 3.23	9	94	76.10 ± 5.60	7	101
**kidney**	48 h at 4 °C in autosampler	7.22 ± 0.53	7	96	36.30 ± 0.78	2	91	73.83 ± 3.25	4	98
	three freeze - thaw cycles	7.24 ± 0.09	1	96	36.70 ± 3.27	9	92	73.93 ± 3.26	4	98
	frozen at −80 °C for 7 days	7.53 ± 0.48	6	100	41.03 ± 2.68	6	102	73.33 ± 3.90	5	98
	frozen at −80 °C for 21 days	7.07 ± 0.18	3	94	44.40 ± 1.13	2	111	71.17 ± 4.84	7	95
**testes**	48 h at 4 °C in autosampler	7.12 ± 0.55	8	95	36.40 ± 0.62	2	91	73.97 ± 3.19	4	99
	three freeze - thaw cycles	7.40 ± 0.44	6	99	37.23 ± 2.90	8	93	72.37 ± 2.15	3	96
	frozen at −80 °C for 7 days	7.27 ± 0.15	2	97	40.93 ± 0.76	2	102	74.17 ± 2.61	3	99
	frozen at −80 °C for 21 days	7.21 ± 0.86	12	96	44.00 ± 0.30	0.7	110	72.93 ± 2.37	3	97
**spleen**	48 h at 4 °C in autosampler	6.91 ± 0.06	1	92	38.47 ± 1.98	5	96	74.33 ± 3.80	5	99
	three freeze - thaw cycles	7.23 ± 0.23	3	96	38.25 ± 1.82	5	95	72.87 ± 2.86	4	97
	frozen at −80 °C for 7 days	7.46 ± 0.26	3	99	40.47 ± 0.95	2	101	74.13 ± 1.68	2	99
	frozen at −80 °C for 21 days	7.62 ± 0.68	9	102	40.37 ± 3.53	9	101	73.30 ± 2.34	3	98

The slopes were calculated from semi-log concentration – time plots using at least 3 data points fitted to a linear regression (Figure S1).

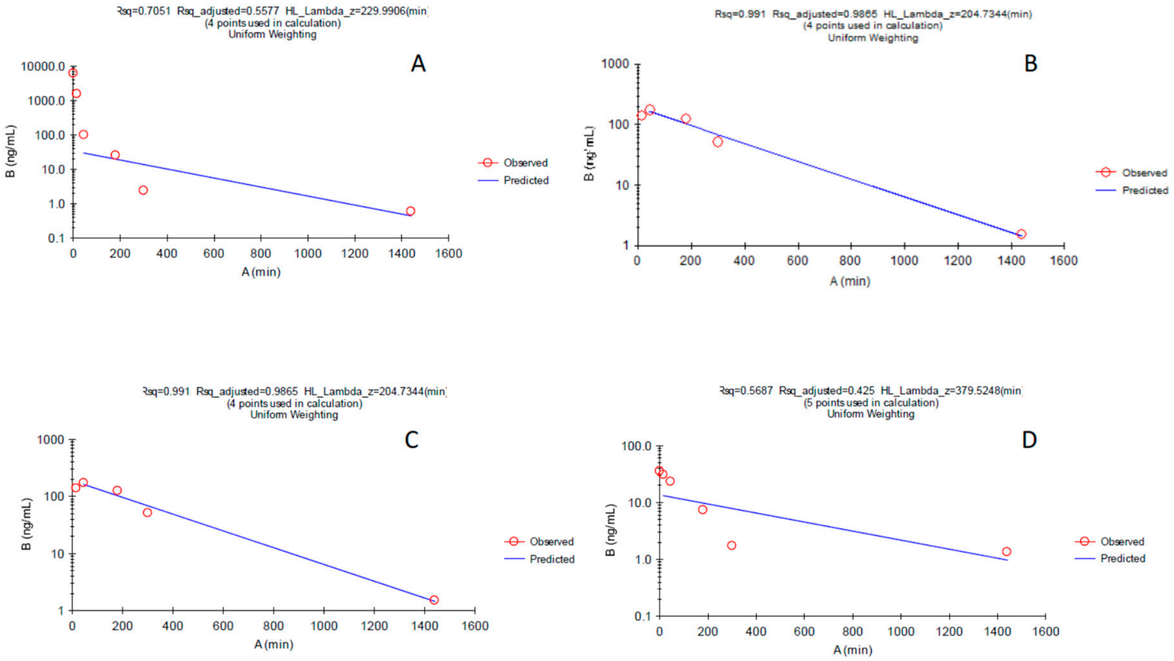

Figure S1. Estimation of elimination rate constants using non-compartmental analysis after i.v. (**A**), i.p. (**B**) administration of DAI suspension, and i.v. (**C**), i.p. (**D**) administration of GCD-EDA/DAI complex (3–5 points were used in calculation).
